# Impact of PEWS on Perceived Quality of Care During Deterioration in Children With Cancer Hospitalized in Different Resource-Settings

**DOI:** 10.3389/fonc.2021.660051

**Published:** 2021-06-23

**Authors:** Marcela Garza, Dylan E. Graetz, Erica C. Kaye, Gia Ferrara, Mario Rodriguez, Dora Judith Soberanis Vásquez, Alejandra Méndez Aceituno, Federico Antillon-Klussmann, Jami S. Gattuso, Belinda N. Mandrell, Justin N. Baker, Carlos Rodriguez-Galindo, Asya Agulnik

**Affiliations:** ^1^ Department of Global Pediatric Medicine, St. Jude Children’s Research Hospital, Memphis, TN, United States; ^2^ Division of Quality of Life and Palliative Care, St. Jude Children’s Research Hospital, Memphis, TN, United States; ^3^ Department of Oncology, Unidad Nacional de Oncología Pediátrica, Guatemala City, Guatemala; ^4^ Department of Nursing, Unidad Nacional de Oncología Pediátrica, Guatemala City, Guatemala; ^5^ Department of Critical Care, Unidad Nacional de Oncología Pediátrica, Guatemala City, Guatemala; ^6^ Francisco Marroquin University School of Medicine, Guatemala City, Guatemala; ^7^ Department of Nursing Research, St. Jude Children’s Research Hospital, Memphis, TN, United States; ^8^ Division of Critical Care, St. Jude Children’s Research Hospital, Memphis, TN, United States

**Keywords:** cancer, critical care, pediatric oncology, early warning systems, clinical deterioration, qualitative analysis

## Abstract

**Background:**

Children with cancer are at high risk for clinical deterioration and subsequent mortality. Pediatric Early Warning Systems (PEWS) have proven to reduce the frequency of clinical deterioration in hospitalized patients. This qualitative study evaluates provider perspectives on the impact of PEWS on quality of care during deterioration events in a high-resource and a resource-limited setting.

**Methods:**

We conducted semi-structured interviews with 83 healthcare staff (nurses, pediatricians, oncology fellows, and intensivists) involved in recent deterioration events at two pediatric oncology hospitals of different resource levels: St. Jude Children’s Research Hospital (SJCRH; n = 42) and Unidad Nacional de Oncología Pediátrica (UNOP; n = 41). Interviews were conducted in the participant’s native language (English or Spanish), translated into English, and transcribed. Transcripts were coded and analyzed inductively.

**Results:**

Providers discussed both positive and negative perspectives of clinical deterioration events. Content analysis revealed “teamwork,” “experience with deterioration,” “early awareness,” and “effective communication” as themes associated with positive perception of events, which contributed to patient safety. Negative themes included “lack of communication,” “inexperience with deterioration,” “challenges with technology”, “limited material resources,” “false positive score,” and “objective tool.” Participants representing all disciplines across both institutions shared similar positive opinions. Negative opinions, however, differed between the two institutions, with providers at UNOP highlighting limited resources while those at SJCRH expressing concerns about technology misuse.

**Conclusion:**

Providers that care for children with cancer find PEWS valuable to improve the quality of hospital care, regardless of hospital resource-level. Identified challenges, including inadequate critical care resources and challenges with technology, differ by hospital resource-level. These findings build on growing data demonstrating the positive impact of PEWS on quality of care and encourage wide dissemination of PEWS in clinical practice.

## Introduction

Pediatric patients with cancer are at risk for clinical deterioration due to multiple factors, including cancer-related complications and treatment-associated toxicities (1). Up to 30 percent of children with cancer will need admission to the intensive care unit (ICU) at least once during their treatment (2). Delays in transfer to the ICU for critically ill pediatric cancer patients are associated with worse outcomes, including higher mortality and end organ dysfunction (1).

Almost 15 years ago, the Institute for Healthcare Improvement identified rapid response systems as a core mechanism to decrease preventable morbidity and mortality (3). To aid in rapid response notification, Pediatric Early Warning Systems (PEWS) are used at many hospitals. PEWS are bedside tools composed of a scoring instrument and an associated intervention algorithm used to proactively identify clinical deterioration and facilitate early transfer to the ICU (4). PEWS have been validated for use with pediatric oncology patients, including in resource limited settings (5–7), and have been shown to reduce frequency of clinical deterioration in hospitalized pediatric cancer patients (8) and improve interdisciplinary communication (9, 10).

Although robust data exist describing the quantitative impact of PEWS on patient outcomes (4), qualitative analyses of the effects of these tools on the perception of quality of delivered care are lacking. Furthermore, provider perceptions of PEWS across hospitals with varying resource levels remain understudied. In this study, we evaluate the impact of PEWS on provider perceptions of care provided to deteriorating pediatric oncology patients at two hospitals of different resource levels.

## Methods

### Research Design and Context

Interviews were conducted during the fall of 2018 at two hospitals dedicated to the care of children with cancer: Unidad Nacional de Oncología Pediátrica (UNOP) in Guatemala City, Guatemala and St. Jude Children’s Research Hospital (St. Jude) in Memphis, USA. Study design and methods have been previously described (10).

St. Jude and UNOP were chosen due to their similar patient volume, size, and recent PEWS implementation (5, 11). In both hospitals, the PEWS scoring tool comprises vital signs, physical exam, treatment requirements, and staff and family concern paired with a response algorithm that defines the next steps in patient care (**Supplemental Figures 1** and **2**).

While similar in organization, the two participating hospitals have different resources. St. Jude employs more than 40 oncologists and 8 intensivists, while UNOP has 9 oncologists and 4 intensivists. The nurse-to-patient ratios in the PICU and inpatient wards are 1:1–1:2 and 1:4–1:6 in UNOP, while they are 1:1 and 1:2, respectively, at St. Jude. The estimated survival rate for children treated in Guatemala, an upper middle-income country, is approximately 65% compared to >80% in the United States, a high-income country. For these reasons, UNOP is considered a resource-limited setting, while St. Jude is considered a high-resource setting.

### Participants

We recruited physicians, advanced practice providers (APP), and nurses at UNOP and St. Jude who were familiar with PEWS and had recently been involved in the care of a patient with a deterioration event (defined as a hospitalized pediatric oncology patient who experienced a clinical worsening requiring an unplanned transfer from the inpatient ward to the ICU). A total of 83 interviews were conducted (**Table 1**) at which point thematic data saturation was reached at each hospital and within each discipline (12).

**Table 1:** Demographics.

	St. Jude n (%)	UNOP n (%)	Worked at St. Jude prior to PEWS implementation n (%)	Worked at UNOP prior to PEWS implementation n (%)
*ICU providers*				
ICU nurse	2 (5)	0 (0)	1 (2)	0 (0)
Advance practice practitioner (NP/PA)	5 (12)	0 (0)	5 (12)	0 (0)
ICU fellow	0 (0)	6 (15)	0	0 (0)
ICU attending physician	6 (14)	1 (2)	5 (12)	1 (2)
Total	*13 (31)*	*7 (17)*	11 (26)	1 (2)
*Floor physicians*				
Oncology fellow	6 (14)	6 (14)	0 (0)	0 (0)
Resident/pediatrician	3 (7)	8 (20)	2 (5)	4 (10)
Advance practice practitioner (NP/PA)	7 (17)	0 (0)	5 (12)	0 (0)
Total	*16 (38)*	*14 (34)*	7 (17)	4 (10)
*Nurses*				
Coordinator	2 (5)	8 (20)	2 (5)	4 (10)
Bedside nurse	11 (26)	12 (29)	9 (2)	7 (17)
Total	*13 (31)*	*20 (49)*	11 (26)	11 (27)
**Total**	**42 (100)**	**41 (100)**	**29 (69)**	**16 (39)**

ICU, intensive care unit; N/A, not available; NP, nurse practitioner; PA, physician assistant; St. Jude, St. Jude Children’s Research Hospital; UNOP, Unidad Nacional de Oncología Pediátrica. Adapted from Graetz D. et al. (10).

### Interview Guide Development and Data Collection

The bilingual research team developed an interview guide to elicit provider perceptions of PEWS and its impact on the care of pediatric oncology patients with recent deterioration events (**Supplemental Figure 3**). Interviews were conducted by a native speaker (MG, JG). The audio from the interviews were recorded and transcribed for analysis. Spanish interviews were professionally translated into English.

### Data Analysis

Transcribed interviews underwent qualitative content analysis using codes derived inductively from transcript review (13). Code definitions are described in **Supplemental Table 1** (10). Each interview transcript was independently coded by two researchers (MG, DG, GF). Transcripts were reviewed by a larger team including a third-party adjudicator to establish consensus and test inter-rater reliability (MG, DG, GF, AA). Segments with overlapping codes of “perception of deterioration event” and “perception of PEWS” were explored. Additional themes were identified and analyzed. An additional subanalysis was conducted using only transcripts of participants who worked at either institution before PEWS implementation. MAXQDA software was used for data management and analysis. COREQ guidelines were followed to ensure rigor of qualitative analysis and manuscript preparation (14).

### Human Subjects

This study was approved by the ethics committee at UNOP and the St. Jude Institutional Review Board.

## Results

### Positive Perception of Care Delivery During Deterioration

Providers described elements that led to the positive perception of deterioration events, including “teamwork” and “experience with deterioration,” while PEWS influenced positive perceptions through “early awareness and timely intervention” and “effective communication” (**Table 2**).

**Table 2:** Positive perception of care delivery during deterioration.

Theme		Provider	Example
Indirect impact of PEWS	Teamwork	Intradisciplinary	
		UNOP Ward Physician	“Because it was late afternoon and many doctors were not present, I went downstairs and one of my colleagues was there helping us.”
		St. Jude Ward Nurse	“There are other nurses checking in on you, ‘Are you okay, can I do something for you? What can I bring you?’”
		Interdisciplinary	
		UNOP Ward Nurse	“Here if there is something that the oncologists have given us confidence is that they have always taken us into account.”
		St. Jude ICU APP	“PEWS gets all the teams together and it makes them talk, and it makes them collaborate.”
	Experience with Deterioration	UNOP Ward Physician	“I think the intensive nursing staff has more experience in ventilating a child than the nurses who are on the ward.”
		St. Jude Ward Physician	“I’ve seen this happen like 15 to 20 times where a patient who is consistently tachycardic without a fever, they are automatically almost near the top of my list because that means they are about to go into hemodynamic instability, it’s just matter of time.”
Direct impact of PEWS	Early Awareness	UNOP ICU Physician	“Patients in general are detected early and in the ICU we have almost no patients detected late that require many more interventions or that the outcome is fatal.”
		St. Jude ICU Physician	“I have personally caught patients early and transferred them to the ICU early, there have been less [Rapid Response Teams] on the floor since it’s been implemented.”
	Effective Communication	UNOP Ward Physician	“It’s a method that helps at least the nursing team to see some … some signs of the patient and communicate to us any anomaly.”
		St. Jude Ward Nurse	“So, I feel like it gave our nurses on the floor a lot of empowerment to say, what I’m seeing and what I’m, you know, assessing, is real, and I’m concerned, and this is my objective data for it.”

St. Jude, St. Jude Children’s Research Hospital; UNOP, Unidad Nacional de Oncología Pediátrica; PEWS, Pediatric Early Warning System; APP, Advance Practice Provider; ICU, Intensive Care Unit.

#### Teamwork

At both hospitals, “teamwork” was a key theme that contributed to positive perceptions of care delivery during deterioration. Participants from all disciplines described how working together helped patient management during these acute events. Ward physicians in both institutions highlighted how their co-workers offered to help when they had critical patients or other urgent tasks: *“We seek to help our team members, if there is a girl that needs to be ventilated, we all support our colleague”* (ward physician, UNOP). Similarly, at St. Jude, physicians mentioned how communication aided decision-making and allowed teams to develop plans together: *“I called the attending, the fellow … and we were all in there so, we decided to go ahead and do push-pull-bolus on him”* (ward physician, St. Jude).

In addition to supporting other providers within their own discipline, participants described how interdisciplinary collaboration improved patient care: “*It was in collaboration: the oncologist who was in charge of the service, the intensivist on duty, the intensive care personnel and the infectious diseases specialist all helped and the treatment decision was taken together”* (ward physician, UNOP).

#### Experience With Deterioration

Respondents’ previous experience with critically ill patients impacted their perceptions of clinical deterioration events: respondents with more experience caring for critical children were more likely to describe positive perspectives. This concept was described similarly at both St. Jude and UNOP: “*So, I think in my case I’ve always taken measures, if not time appropriate, at least in my shift, transfer if required or if not, it is handled in the service but always with quick response*” (ward nurse, UNOP); “*I think it was an appropriate move to have her have him evaluate it, because especially as a young adult your heart rate and respirations don’t have to be too terrible high for you to rank up to a four, so I think it was okay*” (ward APP, St. Jude).

#### Positive Impact of PEWS on Quality of Care

All interviewees spoke positively about the impact of PEWS on the quality of patient care. PEWS were seen to directly improve care delivery through aiding with early clinical assessments, timely interventions, and effective communication between disciplines. Through this, PEWS also indirectly led to improvements in patient care *via* themes associated with positive perceptions of deterioration by encouraging teamwork and helping identify deterioration for those with less experience.

#### Early Awareness and Timely Intervention

In both hospitals, PEWS prompted early evaluation, coded as “early awareness.” Respondents from all disciplines described PEWS as an alarm triggering timely evaluation or intervention. At St. Jude, an ICU provider mentioned: “*Now we seem to be warding off a lot of that and we’re getting a lot more [activations of the rapid response team] and a lot more catching these kids earlier on in their disease process”* (ICU APP, St. Jude). Similar sentiments were seen at UNOP: *“We have not taken care of a respiratory arrest on the floor, since all patients have been transferred on time with the use of PEWS”* (ward nurse, UNOP). Having a system that facilitates and justifies the escalation of patient care was a recurrent concept.

#### Effective Communication

PEWS was also described as enabling interdisciplinary communication, which in turn fostered teamwork and led to positive perceptions of care. Nurses described how PEWS helped them express their concerns to physicians: *“Also it helps us to go with the doctor and have a backup to tell him that this is happening”* (ward nurse, UNOP). Physicians perceived a benefit to the whole care team: *“In a way it empowers staff because many times the staff at the bedside don’t feel like they’re heard”* (ICU physician, St. Jude).

### Negative Perception of Care Delivery During Deterioration

As participants reflected on critical patient events, they also described negative perceptions of care delivery, which focused on lack of communication, inexperience, challenges with technology, and limited material resources, while PEWS influenced negative views due to false positive scores and the objective nature of the tool (**Table 3**).

**Table 3:** Negative perception of care delivery during deterioration.

Theme		Provider	Example
Indirect impact of PEWS	Limited Material Resources	UNOP ICU Physician	“That part of not having a [ICU] bed, I think we were late and maybe we would have done another intervention before.”
	Challenges with Technology	St. Jude ICU APP	“We were finally called to the [PEWS] that morning—and it was kind of a sticky situation because the paging system was down.”
Direct impact of PEWS	False Positive Score	UNOP ICU Physician	“Maybe we will have more alarms that sound and are false alarms, and I know that that in general is an annoying for ICU fellows and even for some ICU attendings.”
		St. Jude ICU Physician	“And just my concern it’s put a lot of workload on the ICU to the point that sometimes it’s the boy who cried wolf and we maybe don’t pay much attention to it like we should.”
	Objective Tool	St. Jude ICU Physician	I think, that’s what I was worried about that people do not use a score, a number rather than a clinical judgment to say, “There’s something wrong with my patient.”
		St. Jude ICU APP	Now I’m seeing it across the board, I even see now, nurses who I deemed experienced in tenure, rely heavily on these systems and I know them previously, and I know they know, you know, better, for lack of a better description.
Lack of Communication		UNOP Ward Physician	“The nurse didn’t communicate to the doctor and the doctor didn’t monitor the patient.”
		St. Jude Ward Physician	“If the nurse doesn’t know to notify the provider, like not being notified that your patient is being transferred to the ICU is really bad.”
Inexperience with Deterioration		UNOP Ward Physician	“Maybe she is a person who doesn’t have too much relation or contact with critical patient and that’s why she is not concerned.”
		St. Jude Ward Physician	“Providers who have more difficulty with that would most likely be the residents because they do not rotate through the ICU as an intern actually, so they don’t have that level of experience of intubating a kid and seeing them on pressors.”

St. Jude, St. Jude Children’s Research Hospital; UNOP, Unidad Nacional de Oncología Pediátrica; PEWS, Pediatric Early Warning System; APP, Advance Practice Provider; ICU, Intensive Care Unit.

#### Lack of Communication

Participants described lacking and ineffective communication during deterioration events leading to negative perceptions of care delivery. In both settings, providers negatively described instances in which physicians were not notified of deteriorating patients. At UNOP a participant stated: *“The nurse didn’t communicate to the doctor and the doctor didn’t monitor the patient”* (ward physician, UNOP). Similar impressions were shared by clinicians at St. Jude: *“A patient had a rapid response called and transferred to the ICU, and the primary provider was never notified”* (ward physician, St. Jude).

#### Inexperience With Deterioration

At both institutions, providers with negative perceptions regarding critical deterioration events often cited lack of experience with patient deterioration as a variable that may contribute to negative perspectives. At UNOP, a physician noted that ward nurses are not used to performing critical interventions: *“For example, all the ventilation and tachycardia, intensive is in charge of that part; ward nurses were not prepared for [clinical deterioration], it had almost never happened, then we brought the nurses from intensive care”* (ward physician, UNOP). A physician at St. Jude mentioned that when new healthcare providers start working in their hospital, they have no experience with deterioration in this patient population, so it takes more time to start the appropriate interventions: *“Some people would have sent this kid to the ICU immediately, but you have a patient needing high levels of care, with a resident who just got out of first year and has no experience with St. Jude in terms of the unique medical knowledge”* (ward physician, St. Jude).

#### Limited Material Resources and Challenges With Technology

Availability or lack of resources influenced the perception of deterioration events and differed by institution. At UNOP, negative perspectives of events focused on the lack of infrastructure impacting the ability of teams to follow the escalation pathway and provide appropriate critical care to deteriorating patients. Specifically, a participant mentioned: *“The patient required intensive care and we hadn’t space in the ICU, so we moved him to intermediate care”* (ward physician, UNOP). At St. Jude, participants did not describe infrastructure limitations. Instead, negative perceptions of deterioration events were linked to challenges caused by technology limitations. For example: *“Another major barrier, and this is kind of a barrier with communication with the ICU, is that the nurse practitioners on-call on the on-call schedule, often the only number listed is not a pager that they have, or a cellphone that they have, but an office number, is an issue”* (ward physician, St. Jude).

#### Negative Impact of PEWS on Quality of Care

While PEWS contributed to positive perceptions of care delivery, 90% of participants also mentioned negative aspects of PEWS. PEWS were perceived to directly contribute to inadequate care delivery by raising false positive alerts and the objective nature of the scoring tool. PEWS indirectly contributed to inadequate care delivery through limited material resources and challenges with technology.

#### False Positive Score

Some participants felt that the PEWS score did not correlate with the clinical impression of the providers, indicating a false positive score, defined as a high PEWS score triggering a rapid response team activation or ICU consult without true need for care escalation. Although false positives were mentioned at both institutions, perceived outcomes secondary to false positives differed between hospitals, causing a sense of alarm fatigue at St. Jude and overuse of resources at UNOP. At St. Jude, participants stated: *“There’s also a sense of, we get patients who for whatever reason, may have a higher score, but clinically are quite stable”* (ICU APP, St. Jude), and *“[It] creates alarm fatigue, because, you know, it’s mild tachycardia, but, there’s absolutely nothing, and really there’s nothing wrong with that heart rate and nothing for us to be concerned about at all”* (ward APP, St. Jude). At UNOP, respondents mentioned receiving consults on patients that have a high score but do not need a critical intervention: *“…Sometimes the consults that I have had are things that … there are other factors … that could affect certain values ​​of the PEWS and maybe it is not a patient that is unstable and that needs an emergency intervention*” (ICU physician, UNOP). Exclusively at UNOP, false positive alerts led to a perception of excessive use of resources: *“It harmed the unit by bringing a girl who did not need to, that was the bad thing and we lost resources”* (ICU physician, UNOP).

#### Objective Tool

The strictly objective nature of the tool, coded as “objective tool,” was viewed negatively at St. Jude, with participants describing PEWS as too automatic: *“Basically, the computer’s doing everything for you and you don’t use your coworkers to help you out”* (ICU APP, St. Jude). An ICU nurse said that it takes away the nurses’ clinical ability to evaluate the patient: *“I don’t think they’re looking at the patient, they’re looking at numbers, numbers, numbers”* and come up with a clinical judgment *“because the scoring system is so objective, I think it takes away a lot of the nurse’s ability to think for themselves”* (ICU APP, St. Jude). This theme was not identified within UNOP interviews.

### Perspective Before and After PEWS Implementation

A subset of participants (54%, n = 45) worked at their respective institutions prior to PEWS implementation, providing insight into the impact of PEWS on patient care (**Table 4**). When reflecting on the quality of patient care pre- and post-PEWS implementation, participants from both institutions primarily described the positive impact of PEWS. They mentioned PEWS impact on early awareness and timely interventions: *“Before, I remember that we transferred patients almost ventilated to intensive, then it was very late … but now if we can say that in its entirety, it may be that one or the other that escape but almost all patients are transferred early*” (ward nurse, UNOP).

**Table 4:** Perspective before and after PEWS implementation.

Provider	Segment
UNOP Ward Nurse	“If I see the two sides of the coin, because I was here before the [PEWS] was implemented, then we did see that it took us so long when we were going to transfer a patient that was complicated to intensive.”
UNOP Ward Nurse	“Compared now is a different world, the complication is detected early, we have been trained on the danger signals for a crash, the [PEWS] has been closely studied.”
UNOP Ward Nurse	“Having implemented the [PEWS] within the unit was gaining importance inside the hospital because we already had an instrument that supported us and we could go to the doctor and tell him the case and one what I didn’t see according to the scale, so one relies on the [PEWS] scale to say the patient is not well.”
UNOP ICU Physician	“I believe that there is a difference that can be seen in the reduction of mortality, there may have an increase in admissions to intensive, but we have a better survival and much shorter vasopressors times and better results after [PEWS] than before [PEWS].”
St. Jude ICU Physician	“They wouldn’t call you till like the patient was actually, we would go to the floor and start like doing CPR, so sometimes, not every time, but sometimes, but the culture now is like from the medicine room to the floors, they call us for whenever they’re concerned.”
St. Jude Ward Physician	“I think, for the most part, it has improved, one, the ICU knowing about these sick patients early”
St. Jude Ward Physician	“Especially for those of us who have been here previously where sometimes kids get way too sick and we don’t know if anything was going on for a long period of time. It’s nice to have sort of a safety net as well.”
St. Jude ICU Physician	“Now if you have an [PEWS] like no one will question you why did you call for ICU consult or for rapid response team because my [PEWS] is high.”
St Jude Ward Nurse	“and it would make you feel very uncomfortable if I ever needed to call again because people made you feel like you were dumb for calling it”
St. Jude ICU APP	“Yeah, I think that it’s helped foster closer relationships between nursing services and us, and giving us a presence that we didn’t have before.”

St. Jude, St. Jude Children’s Research Hospital; UNOP, Unidad Nacional de Oncología Pediátrica; PEWS, Pediatric Early Warning System; APP, Advance Practice Provider; ICU, Intensive Care Unit.

At UNOP, PEWS was described as positively changing patient care and improving safety. Ward nurses stressed that PEWS led them to prioritize vital signs: *“We did not even handle the vital signs, but we had a nursing assistant who wrote down the signs”* (ward nurse, UNOP). In addition, at UNOP, PEWS encouraged nurses to complete a physical assessment on each patient, which was not consistently done previously: *“If you do the [PEWS] you are required to do a total physical examination, maybe before we didn’t do that 100%”* (ward nurse, UNOP).

At St. Jude, ward nurses believed that the implementation of PEWS helped with prioritizing patient care and facilitated activation of emergency response systems. Ultimately, PEWS helped mitigate the nurses’ feelings of criticism for calling a rapid response: *“They’ve kind of calmed down that judgment towards the nurse which helps they understand this is the [PEWS] score this is why we’re being called, it’s not the dumb nurse doesn’t know this or that”* (ward nurse, St. Jude). As a result of these changes, participants felt patient safety improved: “*In the 13 years I’ve been here, it’s probably been the most impactful on patient safety” (*ward nurse, St. Jude).

**Figure 1:**
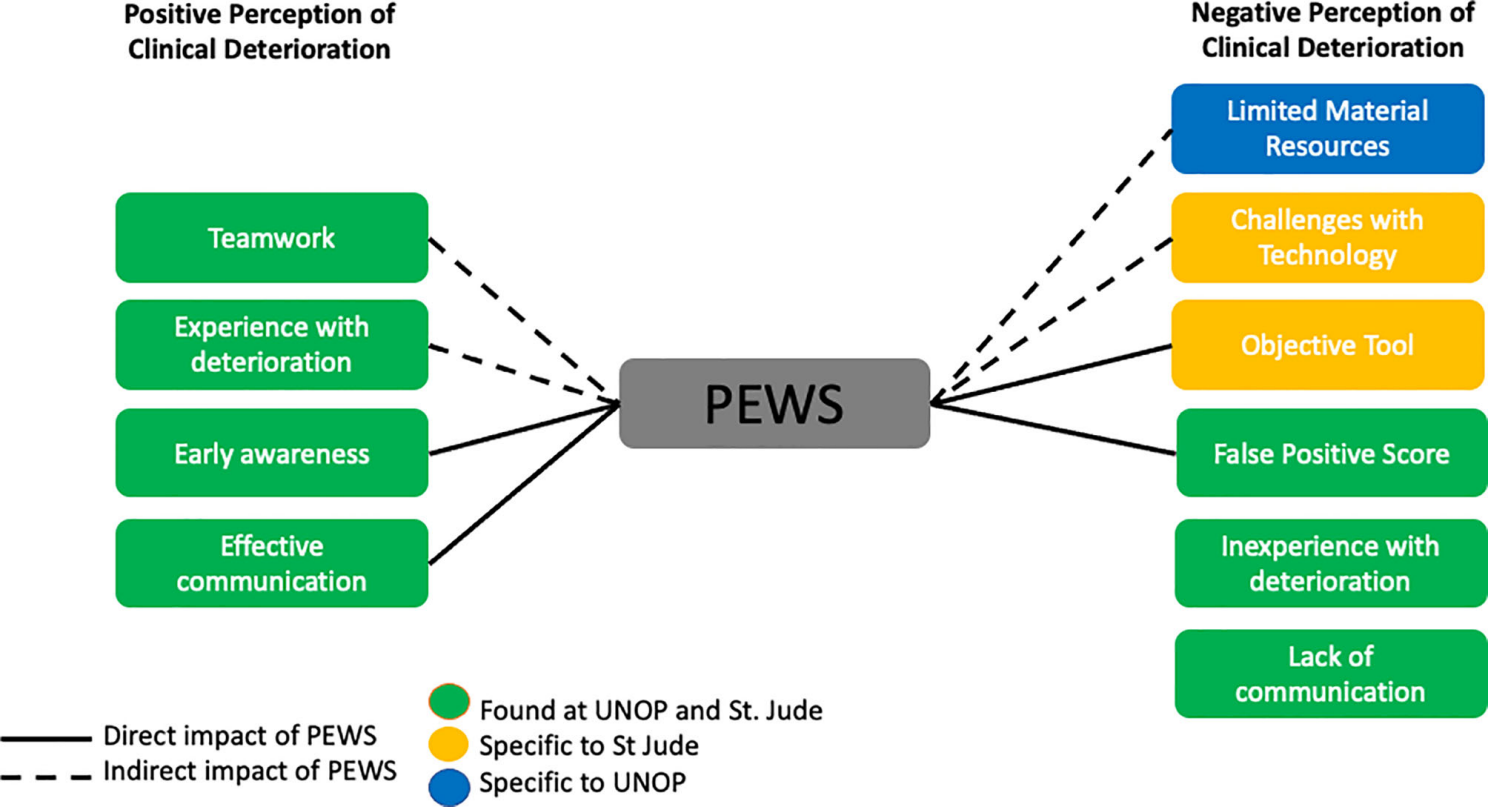
Conceptual Model of Perceptions of Care Delivery During Deterioration.

A model describing our findings regarding provider perceptions of the quality of care delivered during clinical deterioration and the direct and indirect impact of PEWS is summarized in **Figure 1**.

## Discussion

This study builds on the growing understanding of the importance of PEWS in the care of hospitalized children with cancer in settings of varying resource levels. Our study provides additional insights into how providers perceive the impact of PEWS on the quality of care of deteriorating patients, including how it enhances teamwork and effective communication, builds on clinical experience, and promotes early awareness of clinical deterioration.

Despite interviews focused on clinically deteriorating patients, all participants, regardless of discipline or resource setting, mentioned positive characteristics of care delivery in these acute situations. The positive themes concentrated on the perceived efficiency of the team providing care and using PEWS to improve clinical outcomes. Our findings support the idea that PEWS increases interdisciplinary communication by empowering providers and thus improving teamwork, contributing positively to the perception of the quality of care delivered during deterioration events (10). One of the foundations for safe care delivery is effective teamwork, striving toward a common goal (15, 16), and our findings suggest that PEWS can facilitate this phenomenon in the care of deteriorating patients. Furthermore, when effective communication is lacking, teamwork is challenged, leading to worse quality of care and negative provider experience. These findings suggest that additional strategies to optimize team communication can improve the quality of care.

Previous research has shown that PEWS assists with the early identification of deterioration, thereby promoting patient safety and improving outcomes (17). Our findings demonstrate that bedside providers perceive these positive effects during routine clinical care and, specifically, in the moments of critical deterioration. This suggests that staff are invested in the use of PEWS, possibly influenced by its relatively recent implementation. Importantly, our cohort of participants included many providers that worked at these two institutions before the implementation of PEWS. This group concentrated on the positive impact of PEWS on patient care and confirmed the opinion that PEWS promotes patient safety. In addition, PEWS emphasized the role of nursing in patient monitoring, specifically clinical assessment in low resource settings, highlighting the role of nurses in the care of hospitalized pediatric oncology patients (18).

At both institutions, barriers to care escalation existed that were beyond the scope of PEWS: technology at St. Jude and limited resources at UNOP. Although one of the benefits of PEWS is optimization of available resources (19), the lack of infrastructure and staffing continued to be a perceived barrier for ideal care at UNOP. Conversely, in a high-resource setting, technological elements contributed to a negative perception. These resource elements exist outside the reach of PEWS, but nonetheless they should be considered prior to implementation to maximize the impact of PEWS on patient outcomes.

Unsurprisingly, perceptions of deterioration events were influenced by providers’ degrees of clinical experience. Nonetheless, our study suggests that PEWS helps those with less experience to feel more comfortable as it provides an additional aid for patient management. In addition, the objective nature of PEWS is often described as a strength, as it allows for a standard and objective approach to deteriorating patients. Providers in this study did not always view the objectivity of PEWS as positive, pointing to frequent false positive alerts (high PEWS in patients without true deterioration). Although many aspects of PEWS scores are based on objective criteria such as vital signs, the PEWS used at St. Jude and UNOP contains an additional point for both provider and family concern, adding subjectivity to the scoring. Clinician perceptions regarding the value of objective PEWS criteria in contrast to individual clinical knowledge as the most reliable source of decision-making likely impacts the perceived value of PEWS in these settings. While high scores triggering patient evaluation that does not result in escalation of care are inherently needed for the correct use PEWS, this may result in a perceived overuse of critical care resources, especially in settings where these are more limited. Ultimately, for maximum acceptability and efficacy, objective tools should be paired with provider clinical judgement (9).

This study has several limitations. Despite interviews focusing on patients that required unplanned transfer to the ICU, social desirability bias may have prevented people from fully expounding on the negative elements of these events (20). This confounding element was mitigated by having voluntary participation in the study and interviewers external to clinical care teams and hierarchy structures. Although two resource-level settings were chosen as representative of different resource settings, the selected institutions are heavily focused on pediatric cancer care and may not be representative of other hospitals that provide care for children with cancer in either resource setting. Nonetheless, the themes that were discussed are likely applicable beyond these hospital contexts as they are relevant to the clinical care of children with cancer. Also, while interviews were conducted in two languages, Spanish interviews were transcribed and translated to English for analysis in one language. This process may have modified the intended message of the interviewee. To minimize this, a bilingual research team member reviewed a subset of translations prior to their analysis without identified inconsistencies.

In summary, our study shows that providers that care for children with cancer find PEWS valuable for improving quality of care, regardless of hospital resource-level. These findings build on a growing literature supporting the overall positive impact of PEWS on clinical care, thereby encouraging widespread implementation of PEWS in pediatric cancer settings. We also identified shortcomings of PEWS that can be used to improve its use as part of ongoing quality improvement. Further studies, including analysis of the impact of PEWS in other care settings and among different patient populations, will deepen our understanding of the impact of quality improvement interventions and our ability to optimize their use to improve team dynamics and patient outcomes globally.

## Data Availability Statement

The raw data supporting the conclusions of this article will be made available by the authors, without undue reservation.

## Ethics Statement

The studies involving human participants were reviewed and approved by St. Jude Children’s Research Hospital and Unidad Nacional de Oncología Pediátrica. Written informed consent for participation was not required for this study in accordance with the national legislation and the institutional requirements.

## Author Contributions

Conception and design: MG, DG, and AA. Collection and assembly of data: MG, DG, MR, DV, AMA, FA-K, JG, BM, and AA. Data analysis and interpretation: MG, DG, GF, and AA. MG, DG, and AA were responsible for drafting initial manuscript.Accountable for all aspects of the work: All authors. All authors contributed to the article and approved the submitted version.

## Funding

Funded by the American Lebanese Syrian Associated Charities.

## Conflict of Interest

The authors declare that the research was conducted in the absence of any commercial or financial relationships that could be construed as a potential conflict of interest.
