# Supplementary material for: Impact of PEWS on Perceived Quality of Care During Deterioration in Children With Cancer Hospitalized in Different Resource-Settings
Source: Front Oncol. 2021 Jun 23;11:660051. doi: 10.3389/fonc.2021.660051 (PMC8260684; doi:10.3389/fonc.2021.660051)
Supplement: Supplementary file 1 [file DataSheet_1.docx]

**Supplemental Material**

**Impact of PEWS on Perceived Quality of Care During Deterioration in Children with Cancer Hospitalized in Different Resource-Settings**

Marcela Garza, MD^1^, Dylan Graetz, MD^1^, Erica C. Kaye, MD^2^, Gia Ferrara, MSGH^1^, Mario Rodriguez, MD^3^, Dora Judith Soberanis Vásquez, RN^4^, Alejandra Méndez Aceituno, MD^5^, Federico Antillon-Klussmann, MD^3,6^, Jami S. Gattuso, MSN^7^, Belinda N. Mandrell, PhD^7^, Justin N. Baker, MD^2^, Carlos Rodriguez-Galindo, MD^1^, Asya Agulnik, MD^1*^

^1^Department of Global Pediatric Medicine, St. Jude Children’s Research Hospital, Memphis, Tennessee, USA.

^2^ Division of Quality of Life and Palliative Care, St. Jude Children’s Research Hospital, Memphis, Tennessee, USA.

^3^Department of Oncology, Unidad Nacional de Oncología Pediátrica, Guatemala City, Guatemala.

^4^Department of Nursing, Unidad Nacional de Oncología Pediátrica, Guatemala City, Guatemala.

^5^Department of Critical Care, Unidad Nacional de Oncología Pediátrica, Guatemala City, Guatemala.

^6^Francisco Marroquin University School of Medicine, Guatemala City, Guatemala.

^7^Department of Nursing Research, St. Jude Children’s Research Hospital, Memphis, Tennessee, USA.

^7^Division of Critical Care, St. Jude Children’s Research Hospital, Memphis, Tennessee, USA.

**Table of Contents**

| **Item** | **Page** |
| --- | --- |
| Supplemental Table 1. Code Definitions | 2-3 |
| Supplemental Figure 1. Interview Script | 4-5 |
| Supplemental Figure 2. PEWS Scoring Tool | 6 |
| Supplemental Figure 3. PEWS Sample Algorithm | 7 |

***Supplemental Table 1.* Code Definitions**

| **Category** | **Code** | **Definition** |
| --- | --- | --- |
| **Early Awareness** |  | All references to PEWS as a point of EARLY communication increasing provider identification of sick or deteriorating children BEFORE it is too late or significant morbidity/mortality occurs. |
| **Experience** |  |  |
|  | Experience/Inexperience with deterioration | Refers to the familiarity of the healthcare providers with patients that have clinical deterioration. |
| **False Positive Score** |  | References to patients who trigger RRTs because of high PEWS scores but may not really require escalation (e.g. “that kid with the fever who is tachycardic and just needs Tylenol…”) |
| **Lack of Communication** |  | References to when there is an absence of communication (e.g. someone was not notified). Note, this code should be used when communication does not happen, but should not be used when there is communication but specific elements (e.g. a PEWS score) are lacking. This code should also include lack of action (e.g. someone did not complete an order, or did not notify the person they were supposed to about a score). |
| **Objective tool** |  | References to PEWS as a quantitative/objective tool or instrument. This reference may refer to this as a positive or a negative thing, or the reference may be neutral. References to a particular patient's score (PEWS) should not be included e.g. "he scored a 3 for..." References to the score as a "tool" without any mention of it as an objective or specifically quantitative should not be included. |
| **Perception of PEWS** |  |  |
|  | Positive Perception of PEWS | All references to PEWS as a positive tool/instrument. Includes general statements about how PEWS DOES work, but does not include general references to good communication around patient deterioration that is unrelated to PEWS. |
|  | Negative Perception of PEWS | All references to PEWS as a negative tool/instrument. Includes general statements about how PEWS does NOT work, but does not include general references to poor communication around patient deterioration that is unrelated to PEWS. Also does not include general suggestions for additions or alterations to the score. |
| **Perception of Event** |  |  |
|  | Positive Perception of Event | Refers to descriptions of a deterioration event that “went well.” |
|  | Negative Perception of Event | Refers to descriptions of a deterioration event that “did not go well.” |
| **Resources** |  |  |
|  | Limited Material Resources / Challenges with Technology | Includes references to technology (positive, negative or neutral) including medical technology (such as imaging monitors, BiPAP, Hi-flow) and electronic technology (pagers, electronic medical record, automatic alerts) that affect patient care. This also includes elements of infrastructure such as space/beds in a particular unit (e.g. the ICU) or in the hospital. The colored buttons used on the board at UNOP should be coded as technology. Telephone use should not be coded as technology but belongs under "non in-person communication". |
| **Teamwork** |  | References to or descriptions of teamwork, providers coming together and working successfully toward common goal of patient care, or LACK of teamwork. This includes all group communication where providers from more than 2 disciplines are discussing a case together (e.g. primary team + nurse + ICU team). It also includes references to a colleague helping another one (e.g. a nurse covering someone's stable patients during and escalation event, or a nurse covering another nurse's patients while she goes to lunch). Finally, it includes providers not coming together when they should, or coming together and disagreeing. It does not include references to handoff or sign-out which should be coded under "peer communication". |

***Supplemental Figure 1.* Interview Script**

1. Tell me what you think about PEWS
   1. How does it work on your floor/unit?
   2. What is the role of PEWs in patient care?
   3. How does it (or doesn’t it) help you recognize patient deterioration?
   4. What do you like about it?
   5. What don’t you like about it?
   6. What would you change about it?
2. How does PEWs affect communication?
   1. How has it affected your work with...
      1. …nurses and nursing coordinators?
      2. ...residents, fellows and APPs?
      3. ....ICU team members?
      4. ...families?
   2. When you call a provider with a concern, do you include the PEWs score? (or: When a nurse calls you with a concern, do you ask for the PEWs score?)
      1. Why or why not?
      2. How do you decide when to score your concern under “staff concern”?
   3. When is PEWs helpful, when isn’t it?
3. What do you see as the barriers to communication around patient deterioration events?
   1. Can you think of a time when you thought about calling for help/escalating care and did not? (Can you think of a time when you feel care should have been escalated and was not?)?
   2. Why do you think this happened?
4. Were you at St. Jude prior to the implementation of PEWs? If yes....
   1. In what ways do you think it has affected patient safety?
   2. In what ways has it affected/changed communication?
   3. How do you think it has affected the safety culture at St. Jude?
5. Thank you for those thoughts. Now I’d like to talk to you about a specific event, is that okay? I’d like to talk to you about *event* on *day* can you tell me what happened?
   1. What was your shift/assignment like that day? How busy were you/was the team?
   2. What was the patient like? Tell me a bit about their history (e.g. prior deterioration events) and your history with them (did you take care of this patient regularly?)
   3. When did you first realize the patient was deteriorating? What happened next?
   4. Who was called first?
   5. Did the ICU team see the patient before, with, or after the floor providers?
   6. Who talked to the ICU team?
   7. Who made decisions regarding next steps? How were these decisions made? How were the decisions communicated? Were you comfortable with these decisions? Why or why not?
   8. How did the PEWs score help/hurt? How might have this event gone differently without SJAWS?
   9. In your opinion, did this case’s management go well? Is there anything you would have changed or wish had gone differently?
6. Is there anything else you would like to say about PEWs scores and their use here?

***Supplemental Figure 2*. PEWS Scoring Tool**

**
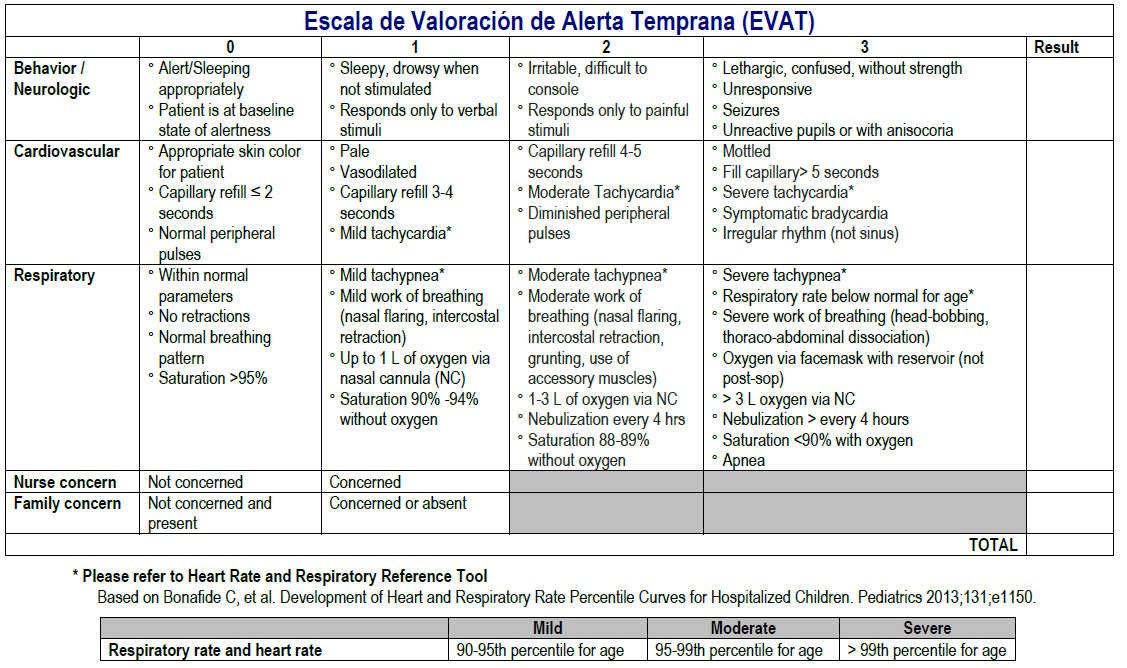
**

***Supplemental Figure 3*. PEWS Sample Algorithm
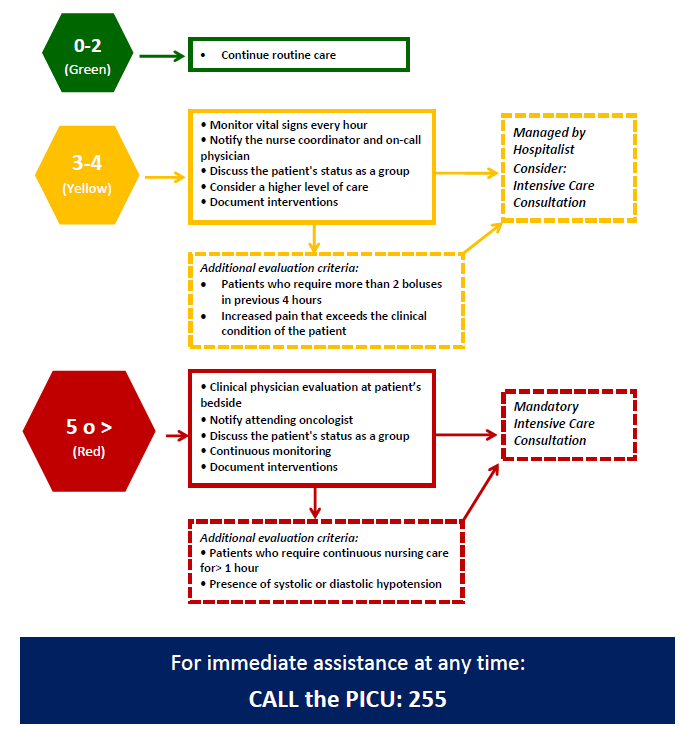
**
